# Experiments in Cataphoresis

**Published:** 1897-01

**Authors:** J. Hall Lewis

**Affiliations:** Dean


### EXPERIMENTS IN CATAPHORESIS.

To the Editor:

Sir,—Inasmuch as cataphoresis is just at present attracting much attention in the dental and medical world, perhaps the following experiments, held in the infirmary of the Columbian University, Dental Department, may prove of interest to the profession.

The apparatus used was that of The Chloride of Silver Dry-Cell Battery Company, of Baltimore, the medicament was thirty per cent. solution (freshly made) of cocaine.

Case I.—Student with compound approximal cavity in first upper molar, decidedly sensitive, and white decay. The dam was applied, a cotton pellet, moistened with the solution cocaine, placed in the dried cavity, the positive pole on the cotton, and the negative at the patient's wrist.

In ten minutes all sensibility was destroyed and the decay removed with the engine-bur, without any attempt at gentleness, the patient feeling no pain whatever.

The cavity was then filled with gold, and during the process of finishing it was noticed that heat from the emery disk caused discomfort, showing a return to normal sensibility.

Case II.—Female, cervico-buccal cavity, shallow and very sensitive; the treatment, time, and results in every way similar to Case I.

Case III.—Female, second lower bicuspid, pulp almost exposed, violent pain for some days. Patient worn out from loss of sleep, etc.

The dam was applied, cavity dried, and the softened substance over pulp gently raised, this being followed by a drop of blood from that organ. The thirty-per-cent. cocaine solution (warmed) on cotton was placed against the pulp, and the electrical current very slowly applied and increased in intensity with like care.

The patient was much frightened at the preparations, and some time was lost in quieting her.

For ten minutes she complained of pain about equal to that of the original ache, but of a different nature; this gradually subsided, and in thirty-five minutes from the time of turning on the current, the pulp was removed entire, with a Donaldson broach, without the patient feeling it in the slightest degree.

(Query: Does not this latter fact prove that the cocaine passes beyond the foramen?)

The root and cavity were at once filled.

J. Hall Lewis, D.D.S.,
*Dean.*

